# Effect of Tai Chi on cardiac function in patients with myocardial infarction: A protocol for a randomized controlled trial

**DOI:** 10.1097/MD.0000000000027446

**Published:** 2021-10-22

**Authors:** ShuJun Zhao, YuGang Zu, Man Lu, XinWei Jia, Xi Chen

**Affiliations:** Affiliated Hospital of Hebei University, Baoding, Hebei, China.

**Keywords:** cardiac function, myocardial infarction, randomized controlled trial, Tai Chi

## Abstract

**Background:**

Myocardial infarction is 1 of the most serious cardiovascular diseases. Early interventional therapy preserves the cardiac function of patients with myocardial infarction to the greatest extent, but it is far from meeting people's need only limited to cardiac revascularization. It is also necessary to help patients improve their quality of life, exercise tolerance, and reduce the incidence of acute cardiac recurrence as much as possible. All these depend on cardiac rehabilitation (CR) are based on exercise. Early and correct CR helps to improve the patient's heart function and improve living standards. Traditional Chinese exercise Tai Chi as an alternative form of CR has gradually become popular, but it lacks large samples and high-quality clinical studies to verify it. This study aims to explore the effect of Tai Chi on the cardiac function of patients with myocardial infarction, and to provide a strong basis for patients to choose which CR exercise.

**Methods:**

This is a prospective randomized controlled trial. 272 patients with myocardial infarction will be randomly divided into an experimental group and a control group according to 1:1, with 136 cases in each group. The control group: conventional treatment; the experimental group: increase Tai Chi exercise on the basis of the control group. Both groups will receive standard treatment for 24 weeks and will be followed up for 3 months. Observation indicators include: total effective rate, 6 minutes walking test, brain natriuretic peptide, left ventricular ejection fraction, the adverse reaction rate, etc. The data will be analyzed by using SPSS 25.0 software.

**Discussion:**

This study will evaluate the effect of Tai Chi on the cardiac function of patients with myocardial infarction. The results of this test will provide clinical evidence for patients to choose which CR exercise.

**Trial registration:**

OSF Registration number: DOI 10.17605/OSF.IO/QKWDP.

## 1 Introduction

With the change in lifestyles, hypertension, hyperglycemia, hyperlipidemia, smoking, lack of exercise, aging of population, and other risk factors, the incidence of coronary heart disease is increasing year by year, and it has become 1 of the main diseases that affect global health.^[1]^ Myocardial infarction is 1 of the cardiovascular diseases with the highest mortality,^[2]^ and it is a serious threat to people's life and health.^[3]^ Early intervention and standardized drug treatment has greatly reduced the mortality of patients with myocardial infarction, but there is a lack of attention to rehabilitation after the onset of disease. Most patients with myocardial infarction fear exercise, resulting in decreased activity tolerance, decreased cardiopulmonary function, anxiety, decreased quality of life, relapsed illness, and often require hospitalization, which brings a heavy burden to the patient's family and society.^[4]^ Therefore, there is an urgent need to find a method that is simple and easy to operate, with less side effects, that can improve patients’ cardiac function and exercise tolerance, thereby improving the quality of life.

Tai Chi, originated in ancient China, is mainly divided into 5 schools: Chen, Yang, Wu, Wu, and Sun. It is divided into 24 styles, 36 styles, and 108 styles. It is a medium-strength defensive exercise,^[5]^ with the characteristics of health care, economy and convenience.^[6,7]^ Studies have found that Tai Chi exercise can increase patient endurance, balance and sensitivity, and improve the quality of life of patients.^[8,9]^ At the same time, moderate-intensity aerobic exercise is the core content of cardiac rehabilitation, which can not only enhance the relative adaptability of heart function, but also improve the blood supply capacity of the coronary artery of the heart to a certain extent, and reduce the cardiovascular mortality rate by 20% to 30%.^[10]^ As an ancient exercise training, Tai Chi has the characteristics of ease and softness, smoothness and unobstructed intention, which is convenient for patients’ participation and persistence. It has advantages for the rehabilitation of patients with myocardial infarction, but there is currently a lack of large sample comparisons in the academic community.

Therefore, we plan to evaluate the efficacy and safety of Tai Chi on the cardiac function of patients with myocardial infarction through this randomized controlled trial.

## 2 Methods

### 2.1 Study design

**Figure 1:**
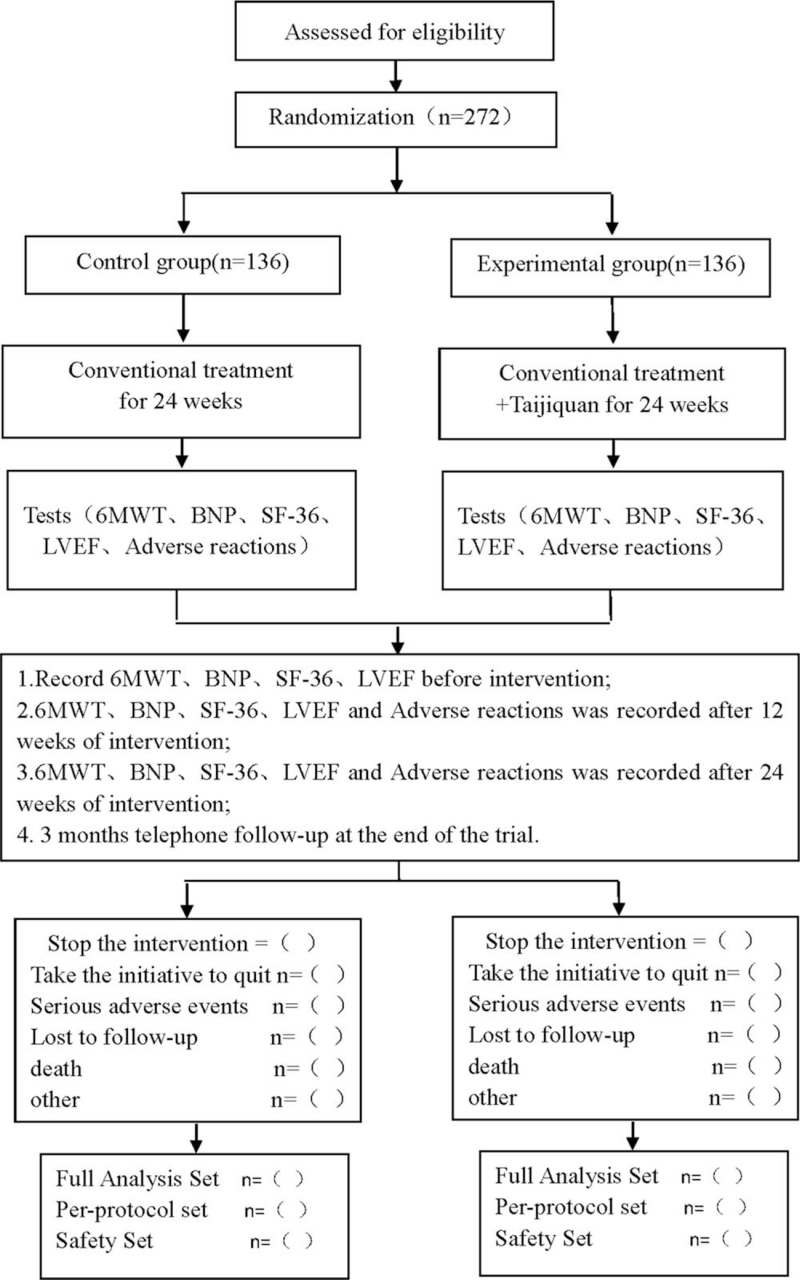
Flow diagram of study. 6MWT = 6 minutes walking test, BNP = brain natriuretic peptide, LVEF = left ventricular ejection fraction, SF-36 = short-form36.

This is a prospective randomized controlled clinical trial to study the effect of Tai Chi on the cardiac function of patients with myocardial infarction. This research protocol follows the latest consolidated standards of reporting trials 2017 (See Fig. 1 for the flow chart), and Standard Protocol Items: Recommendations for Interventional Trials 2013 statement (see SPIRIT checklist, Table S1, Supplemental Digital Content).

### 2.2 Ethics and registration

This research protocol complies with the Declaration of Helsinki and was approved by the clinical research ethics committee of our hospital. This study has been registered in open science framework (OSF) with registration number of DOI 10.17605/OSF.IO/QKWDP. Before randomization, all patients need to sign a written informed consent and they are free to choose whether to continue the trial at any time.

### 2.3 Patients

Diagnostic criteria: ① Diagnosis of myocardial infarction in the fourth edition of the General Definition of Myocardial infarction in the European Society of Cardiology 2018^[11]^; ② According to the New York Heart Association (NYHA) cardiac function classification standard, cardiac function is grade I to III.

Inclusion criteria: ① Meet the diagnostic criteria for myocardial infarction; ② Age ≥35 years old and ≤80 years old; ③ Cardiac function grade I to III; ④ Stable condition after interventional surgery without serious complications; ⑤ Have a certain communication and understanding ability, express their wishes correctly; ⑥ After informed consent and signed an informed consent form, they voluntarily participated in this research project.

Exclusion criteria: (A standard for judging whether a patient can become a experimental object): ① Suffer from acute systemic disease; ② Cardiac function grade IV; ③ Risk stratification of cardiac rehabilitation in patients with coronary heart disease^[12]^ showed that they are at high-risk; ④ Relative or absolute contraindications for rehabilitation exercise such as blood pressure drop during exercise; ⑤ Limb dysfunction; ⑥ Complicated with severe mental illness; ⑦ Unable to understand the research plan after explanation or unwilling to participate.

Exclusion criteria: (A criterion used to screen patients who have become experimental subjects, but the relevant data cannot be included in the final data analysis): ① Severe adverse events or serious complications are not suitable for the next test; ② Poor compliance, which affects the judgment of efficacy and safety; ③ The disease progresses during the treatment process, and the treatment plan needs to be changed; ④ For any reason, the subject asked to withdraw from the trial.

### 2.4 Sample size

According to the calculation formula:


$${\text{n}}_{1}=\pi (1-\pi ){\left[\frac{\left(\mu_{1-\alpha }+\mu_{1-\beta }\right)}{\delta }\right]}^{2}\frac{\left(1+\text{c}\right)}{\text{c}},{\text{n}}_{2}=\text{c}{\text{n}}_{1}$$

Using the superiority test, $\mu_{1-\alpha }=1.64,\mu_{1-\beta }=1.28.\pi =0.8,c=1,\delta =0.15$, calculate n1 = n2 = 122. Considering the clinical dropout rate of about 10%, a total of 272 cases need to be included. The included patients will be numbered according to the order of visits, and will be divided into an experimental group and a control group using a completely random method, with 136 cases in each group.

### 2.5 Interventions

This study will select patients who meet the criteria of this study by recruiting in the hospital. A randomized controlled study will be used, in which the control group: conventional treatment; the experimental group: Tai Chi exercise will be added to the control group. The 2 groups of patients will receive the same routine care. During the trial period, avoid smoking, alcohol and irritating food, avoid strenuous exercise, and pay attention to adverse reactions. When necessary, the attending doctor can appropriately adjust the plan according to the patient's condition, and all intervention methods will be recorded in detail for the final result analysis. Efficacy assessors are not aware of the study group plan, and data statisticians are not involved in the design and implementation of the study. Before and after treatment, the health status of each patient will be evaluated, including the outcome indicators, and all patients will be followed up by telephone. Follow-up content includes cardiovascular events and rehospitalization.

Control group: according to the relevant guidelines after myocardial infarction intervention, standard drug treatments will be given, including antiplatelet drugs, statins, angiotensin converting enzyme inhibitors, nitrates, β-receptor blockers, and health education. The content of the education includes the control of myocardial infarction risk factors, techniques for improving sleep quality, diet guidance, etc, and pays attention to communicating with patients, mastering their psychological and emotional changes, and avoiding or alleviating bad moods such as anxiety and depression in elderly patients.

Experimental group: on the basis of conventional treatment, the experimental group will be given 24 simplified Tai Chi. Professional Tai Chi teachers will teach and practice the subjects to ensure that the practice time and movement rhythm are basically the same. The whole set of Tai Chi includes 3 parts: preparation activity, training activity and end activity, totaling about 20 minutes, twice a day, once in the morning and once in the afternoon, and lasts for 24 weeks.

### 2.6 Outcomes

#### 2.6.1 Efficacy indicators

Main efficacy indicators: total effective rate: referring to the “Guiding Principles for Clinical Research of New Chinese Medicines”,^[13]^ total effective rate = (marked effective number + effective number)/total number × 100%. According to NYHA grading method, evaluate the curative effect of cardiac function: ① Marked effective: cardiac function improved by more than grade 2; ② Effective: cardiac function improved by grade 1, but not as good as grade 2; ③ Ineffective: cardiac function improved less than grade 1; ④ Deterioration: the deterioration of heart function is less than grade 1.

Secondary efficacy indicators: 6 minutes walking test, brain natriuretic peptide, left ventricular ejection fraction, medical outcomes study short-form36 (SF-36).

#### 2.6.2 Adverse reaction rate

Including any uncomfortable symptoms (such as dizziness, nausea, etc) during treatment.

### 2.7 Sample collection

Data will be collected according to the evaluation criteria before the start of treatment, 12 weeks after the treatment, and at the end of 24 weeks of treatment. After the treatment, each patient will be followed up by telephone for 3 months. Detailed follow-up information can not be collected and the reasons for loss to follow-up will be recorded. Access to the database is limited to the researchers of this research group.

### 2.8 Study quality control

Throughout the experiment, each participant will be monitored for safety. All adverse events will be transferred to the clinical trial institution ethics committee of the office, who will review the event and rule on causality. We set up a Data and Safety Monitoring Board (DSMB). Members of DSMB include physicians, clinical pharmacists, test method experts, statistical experts, and members of the ethics committee, who will conduct a risk assessment and safety analysis procedures based on termination conditions.

### 2.9 Statistical analysis plan

The database is established by Excel. The full analysis set refers to the intent-to-treat analysis of all subjects randomly enroll, treat at least once and visit at least once. Per-protocol set: includes all subjects who comply with the trial protocol, have no missing baseline variables, and can measure the main variables. safety set: including random enrollment, at least 1 medication, and at least 1 safety visit after medication.

In this study, SPSS25.0 statistical analysis software will be used for data analysis. If the measurement data meet the normal distribution, the independent sample *t* test is used between the groups, and the paired sample *t* test is used within the group. The results are represented by ($\bar{X}\pm S$); Nonparametric test will be used for those who did not meet the normality, and the results will be expressed in quartiles; Chi square test will be used for counting data. The incidence of adverse events will be compared by chi square test. *P* *<* *.05*, with statistical significance.

## 3 Discussion

The rapid development of interventional technology has significantly improved the treatment effect of patients with myocardial infarction and reduced the mortality rate. However, medical treatment and cardiac revascularization cannot completely solve the problems of rehospitalization and quality of life of patients with myocardial infarction. In patients with myocardial infarction, myocardial cells are repeated ischemic and hypoxic for a long time, cardiac output decreases, ventricular remodeling, heart failure, and death can occur in severe cases.^[14,15]^ The results of a study show that drinking, smoking, low vegetable intake, low exercise, and high psychological stress are important risk factors that affect the occurrence of myocardial infarction on a global scale.^[16]^ A healthy lifestyle and diet can prevent and control myocardial infarction.^[17]^ Therefore, prevention and recanalization of blocked blood vessels is the key to the treatment of myocardial infarction, and the earlier the period of cardiac rehabilitation, the greater the benefit.^[18,19]^ Tai Chi is an economical, simple and convenient form of cardiac rehabilitation that can be exercised at home. It has gradually become a popular trend.

Tai Chi is a traditional boxing skill integrating internal and external cultivation and fighting to strengthen the body. Theoretically, it adheres to the yin-yang syndrome differentiation of ancient Chinese philosophy and the Taoist concept of “Tai Chi”, and also coincides with the Yin-Yang and 5 elements and meridian theory of traditional Chinese medicine. When exercising, hardness and softness are combined with movement and static. It can not only fight the enemy for self-defense, but also cultivate body and mind. It has the functions of relaxing meridians, promoting qi and activating blood circulation. Scholars have found that Tai Chi exercise for 2 years in patients with hypertension and hyperlipidemia can effectively control blood pressure and blood lipid.^[20]^ Practicing Tai Chi in patients with heart disease can help restore aerobic endurance,^[21]^ improve myocardial contraction strength, improve heart blood pumping function, and comprehensively regulate heart and lung by improving respiratory circulation.^[22]^ At the same time, Tai Chi is safe for stable elderly patients with congestive heart failure (NYHA II), and exercises exercise ability and muscle strength at the same time.^[23]^ Compared with other exercise or no exercise, Tai Chi can better improve the quality of life and depression of such patients.^[24,25]^ It is a recommended rehabilitation exercise method.^[26]^

This randomized controlled trial is to verify that Tai Chi can improve the cardiac function of patients with myocardial infarction. Since there is no standard large-sample clinical study to evaluate the effect of Tai Chi on the cardiac function of patients with myocardial infarction, we plan to evaluate its efficacy and safety through prospective randomized controlled studies.

This study also has some limitations: the follow-up time is short, and we cannot understand the impact of long-term efficacy. Therefore, we may extend the follow-up time if necessary.

## Author contributions

**Data collection:** ShuJun Zhao and YuGang Zu.

**Formal analysis:** YuGang Zu and Xi Chen.

**Funding support:** Xi Chen.

**Investigation:** ShuJun Zhao and Man Lu.

**Software operating:** XinWei Jia and Man Lu.

**Supervision:** YuGang Zu and Man Lu.

**Writing – original draft:** ShuJun Zhao and YuGang Zu.

**Writing – review & editing:** ShuJun Zhao and XinWei Jia.

## Supplementary Material

Supplemental Digital Content
