# Supplementary material for: Effect of Tai Chi on cardiac function in patients with myocardial infarction: A protocol for a randomized controlled trial
Source: Medicine (Baltimore). 2021 Oct 22;100(42):e27446. doi: 10.1097/MD.0000000000027446 (PMC8542151; doi:10.1097/MD.0000000000027446)
Supplement: Supplemental Digital Content [file medi-100-e27446-s001.docx]

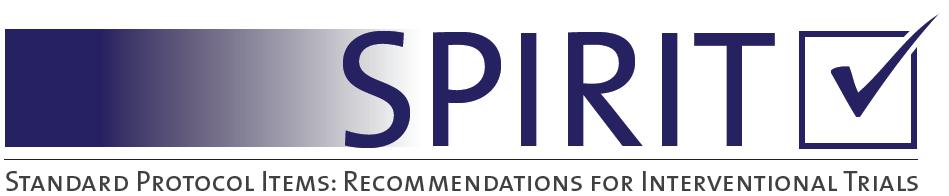


SPIRIT 2013 Checklist: Recommended items to address in a clinical trial protocol and related documents*

| **Section/item** | **Item** | **Description** | **Addressed on** |  | |
| --- | --- | --- | --- | --- | --- |
|  | **No** |  | **page number** |  | |
|  | |  |  | |  |
| **Administrative information** | |  |  | |  |
| Title | 1 | Descriptive title identifying the study design, population, interventions, and, if applicable, trial acronym | Page 1 |  | |
| Trial registration | 2a | Trial identifier and registry name. If not yet registered, name of intended registry | Page 2 |  | |
|  | 2b | All items from the World Health Organization Trial Registration Data Set | Page 2 |  | |
| Protocol version | 3 | Date and version identifier | Page 2 |  | |
| Funding | 4 | Sources and types of financial, material, and other support | Page 1 |  | |
| Roles and | 5a | Names, affiliations, and roles of protocol contributors | Page 9 |  | |
| responsibilities | 5b | Name and contact information for the trial sponsor | Page 9 |  | |
|  |  |  |  |  | |
|  | 5c | Role of study sponsor and funders, if any, in study design; collection, management, analysis, and |  |  | |
|  |  | interpretation of data; writing of the report; and the decision to submit the report for publication, including | Page 9 |  | |
|  |  | whether they will have ultimate authority over any of these activities |  |  | |
|  | 5d | Composition, roles, and responsibilities of the coordinating centre, steering committee, endpoint | Page 9 |  | |
|  |  | adjudication committee, data management team, and other individuals or groups overseeing the trial, if |  |  | |
|  |  | applicable (see Item 21a for data monitoring committee) |  |  | |

1

| **Introduction** |  |  |  |
| --- | --- | --- | --- |
| Background and | 6a | Description of research question and justification for undertaking the trial, including summary of relevant | Page 2 |
| rationale |  | studies (published and unpublished) examining benefits and harms for each intervention |  |
|  | 6b | Explanation for choice of comparators | Page 2 |
| Objectives | 7 | Specific objectives or hypotheses | Page 2 |
| Trial design | 8 | Description of trial design including type of trial (eg, parallel group, crossover, factorial, single group), |  |
|  |  | allocation ratio, and framework (eg, superiority, equivalence, noninferiority, exploratory) | Page 3-4 |
| **Methods: Participants, interventions, and outcomes** | | |  |
| Study setting | 9 | Description of study settings (eg, community clinic, academic hospital) and list of countries where data will | Page 3 |
|  |  | be collected. Reference to where list of study sites can be obtained |  |
| Eligibility criteria | 10 | Inclusion and exclusion criteria for participants. If applicable, eligibility criteria for study centres and | Page 4 |
|  |  | individuals who will perform the interventions (eg, surgeons, psychotherapists) |  |
| Interventions | 11a | Interventions for each group with sufficient detail to allow replication, including how and when they will be | Page 5-6 |
|  |  | administered |  |
|  | 11b | Criteria for discontinuing or modifying allocated interventions for a given trial participant (eg, drug dose | Page 5-6 |
|  |  | change in response to harms, participant request, or improving/worsening disease) |  |
|  | 11c | Strategies to improve adherence to intervention protocols, and any procedures for monitoring adherence | Page 5-6 |
|  |  | (eg, drug tablet return, laboratory tests) |  |
|  | 11d | Relevant concomitant care and interventions that are permitted or prohibited during the trial | Page 5-6 |
| Outcomes | 12 | Primary, secondary, and other outcomes, including the specific measurement variable (eg, systolic blood |  |
|  |  | pressure), analysis metric (eg, change from baseline, final value, time to event), method of aggregation (eg, | Page 6 |
|  |  | median, proportion), and time point for each outcome. Explanation of the clinical relevance of chosen |  |
|  |  | efficacy and harm outcomes is strongly recommended |  |
| Participant timeline | 13 | Time schedule of enrolment, interventions (including any run-ins and washouts), assessments, and visits for | none |
|  |  | participants. A schematic diagram is highly recommended (see Figure) |  |

2

| Sample size | 14 | Estimated number of participants needed to achieve study objectives and how it was determined, including | Page 5 |
| --- | --- | --- | --- |
|  |  | clinical and statistical assumptions supporting any sample size calculations |  |
| Recruitment | 15 | Strategies for achieving adequate participant enrolment to reach target sample size | Page 5 |

**Methods: Assignment of interventions (for controlled trials)**

| Allocation: |  |  |  |
| --- | --- | --- | --- |
| Sequence | 16a | Method of generating the allocation sequence (eg, computer-generated random numbers), and list of any | Page 5 |
| generation |  | factors for stratification. To reduce predictability of a random sequence, details of any planned restriction |  |
|  |  | (eg, blocking) should be provided in a separate document that is unavailable to those who enrol participants |  |
|  |  | or assign interventions |  |
| Allocation | 16b | Mechanism of implementing the allocation sequence (eg, central telephone; sequentially numbered, | Page 5 |
| concealment |  | opaque, sealed envelopes), describing any steps to conceal the sequence until interventions are assigned |  |
| mechanism |  |  |  |
| Implementation | 16c | Who will generate the allocation sequence, who will enrol participants, and who will assign participants to | Page 5-6 |
|  |  | interventions |  |
| Blinding (masking) | 17a | Who will be blinded after assignment to interventions (eg, trial participants, care providers, outcome | none |
|  |  | assessors, data analysts), and how |  |
|  | 17b | If blinded, circumstances under which unblinding is permissible, and procedure for revealing a participant’s | none |
|  |  | allocated intervention during the trial |  |
| **Methods: Data collection, management, and analysis** | | |  |
| Data collection | 18a | Plans for assessment and collection of outcome, baseline, and other trial data, including any related | Page 6 |
| methods |  | processes to promote data quality (eg, duplicate measurements, training of assessors) and a description of |  |
|  |  | study instruments (eg, questionnaires, laboratory tests) along with their reliability and validity, if known. |  |
|  |  | Reference to where data collection forms can be found, if not in the protocol |  |
|  | 18b | Plans to promote participant retention and complete follow-up, including list of any outcome data to be | Page 6 |
|  |  | collected for participants who discontinue or deviate from intervention protocols |  |

3

| Data management | 19 | Plans for data entry, coding, security, and storage, including any related processes to promote data quality | Page 7 |
| --- | --- | --- | --- |
|  |  | (eg, double data entry; range checks for data values). Reference to where details of data management |  |
|  |  | procedures can be found, if not in the protocol |  |
| Statistical methods | 20a | Statistical methods for analysing primary and secondary outcomes. Reference to where other details of the | Page 6 |
|  |  | statistical analysis plan can be found, if not in the protocol |  |
|  | 20b | Methods for any additional analyses (eg, subgroup and adjusted analyses) | Page 7 |
|  | 20c | Definition of analysis population relating to protocol non-adherence (eg, as randomised analysis), and any |  |
|  |  | statistical methods to handle missing data (eg, multiple imputation) | Page 7 |
| **Methods: Monitoring** | |  |  |
| Data monitoring | 21a | Composition of data monitoring committee (DMC); summary of its role and reporting structure; statement of | Page 9 |
|  |  | whether it is independent from the sponsor and competing interests; and reference to where further details |  |
|  |  | about its charter can be found, if not in the protocol. Alternatively, an explanation of why a DMC is not |  |
|  |  | needed |  |
|  | 21b | Description of any interim analyses and stopping guidelines, including who will have access to these interim | Page 7 |
|  |  | results and make the final decision to terminate the trial |  |
| Harms | 22 | Plans for collecting, assessing, reporting, and managing solicited and spontaneously reported adverse | Page 7 |
|  |  | events and other unintended effects of trial interventions or trial conduct |  |
| Auditing | 23 | Frequency and procedures for auditing trial conduct, if any, and whether the process will be independent | none |
|  |  | from investigators and the sponsor |  |
| **Ethics and dissemination** | |  |  |
| Research ethics | 24 | Plans for seeking research ethics committee/institutional review board (REC/IRB) approval | Page 4 |
| approval |  |  |  |
| Protocol | 25 | Plans for communicating important protocol modifications (eg, changes to eligibility criteria, outcomes, | Page 4 |
| amendments |  | analyses) to relevant parties (eg, investigators, REC/IRBs, trial participants, trial registries, journals, |  |
|  |  | regulators) |  |

4

| Consent or assent | 26a | Who will obtain informed consent or assent from potential trial participants or authorised surrogates, and | Page 4 |
| --- | --- | --- | --- |
|  |  | how (see Item 32) |  |
|  | 26b | Additional consent provisions for collection and use of participant data and biological specimens in ancillary | none |
|  |  | studies, if applicable |  |
| Confidentiality | 27 | How personal information about potential and enrolled participants will be collected, shared, and maintained | Page 7 |
|  |  | in order to protect confidentiality before, during, and after the trial |  |
| Declaration of | 28 | Financial and other competing interests for principal investigators for the overall trial and each study site | Page 1 |
| interests |  |  |  |
| Access to data | 29 | Statement of who will have access to the final trial dataset, and disclosure of contractual agreements that | Page 6 |
|  |  | limit such access for investigators |  |
| Ancillary and post- | 30 | Provisions, if any, for ancillary and post-trial care, and for compensation to those who suffer harm from trial | none |
| trial care |  | participation |  |
| Dissemination policy | 31a | Plans for investigators and sponsor to communicate trial results to participants, healthcare professionals, | none |
|  |  | the public, and other relevant groups (eg, via publication, reporting in results databases, or other data |  |
|  |  | sharing arrangements), including any publication restrictions |  |
|  | 31b | Authorship eligibility guidelines and any intended use of professional writers | none |
|  | 31c | Plans, if any, for granting public access to the full protocol, participant-level dataset, and statistical code | none |
| **Appendices** |  |  |  |
| Informed consent | 32 | Model consent form and other related documentation given to participants and authorised surrogates | none |
| materials |  |  |  |
| Biological | 33 | Plans for collection, laboratory evaluation, and storage of biological specimens for genetic or molecular | none |
| specimens |  | analysis in the current trial and for future use in ancillary studies, if applicable |  |

*It is strongly recommended that this checklist be read in conjunction with the SPIRIT 2013 Explanation & Elaboration for important clarification on the items. Amendments to the protocol should be tracked and dated. The SPIRIT checklist is copyrighted by the SPIRIT Group under the Creative Commons “[Attribution-NonCommercial-NoDerivs 3.0 Unported](http://www.creativecommons.org/licenses/by-nc-nd/3.0/)” license.

5
